# Supplementary material for: Comparative analysis of new, mScarlet-based red fluorescent tags in Caenorhabditis elegans
Source: bioRxiv. 2024 Jun 13:2024.06.11.598534. Preprint. [Version 1] doi: 10.1101/2024.06.11.598534 (PMC11195195; doi:10.1101/2024.06.11.598534)
Supplement: Supplement 1 [file NIHPP2024.06.11.598534v1-supplement-1.pdf]

bioRxiv preprint doi: https://doi.org/10.1101/2024.06.11.598534; this version posted June 13, 2024. The copyright holder for this preprint (which was not certified by peer review) is the author/funder, who has granted bioRxiv a license to display the preprint in perpetuity. It is made available under aCC-BY 4.0 International license.

Supplemental file S1: Fluorophore sequences

The *C. elegans* codon-optimized mScarlet-3 and mScarlet-I3 sequences below have been cloned into the pPD95.75 backbone in place of GFP and will be deposited to Addgene with the following plasmid names:

pWXC077: mScarlet3
pWXC078: mSarlet-I3

Overhang sequences used to replace GFP in pPD95.75 were:

5'-gacccttggagggtaccggtagaaaaa-3'
5'-cattcgtagaattccaactg-3'

XXXX coding sequence
xxxx introns

>mScarlet3
ATGGACTCCACCGAGGCCGTCATCAAGGAGTTCATGCGTTTCAAGGTCCACATGGAGGATCCA
TGAACGGACACGAGTTCGAGATCGAGGGAGAGGGAGAGGGACGTCCATACGAGGGAACCCAAAC
CGCCAAGCTCCGTGTCACCAAGgtaagtttaaacatatatatactaactaaccctgattattta
aattttcag**GGAGGACCACTCCCATTCTCCTGGGACATCCTCTCCCCACAATTCATGTACGGAT**
CCCGTGCCTTCACCAAGCACCCAGCCGACATCCCAGACTACTGGAAGCAATCCTTCCCAGAGGG
ATTCAAGTGGGAGCGTGTCATGAACTTCGAGGACGGAGGAGCCGTCTCCGTCGCCCAAGACACC
TCCCTCGAGGACGGAACCCTCATCTACAAGGTCAAGCTCCGTGGAACCAACTTCCCACCAGACG
GACCAGTCATGCAAAAGAAGACCATGGGATGGGAGGCCTCCACCGAGCGTCTCTACCCAGAGGA
CGTCGTCCTCAAGgtaagtttaaacagttcggtactaactaaccatacatatttaaattttcag
GGAGACATCAAGATGGCCCTCCGTCTCAAGGACGGAGGACGTTACCTCGCCGACTTCAAGACCA
CCTACCGTGCCAAGAAGCCAGTCCAAATGCCAGGAGCCTTCAACATCGACCGTAAGCTCGACAT
CACCTCCCACAACGAGGACTACACCGTCGTCGAGCAATACGAGCGTTCCGTCGCCCGTCACTCC
ACCGGAGGATCCGGAGGATCCTAA

>mScarlet-I3
ATGGACTCCACCGAGGCCGTCATCAAGGAGTTCATGCGTTTCAAGGTCCACATGGAGGATCCA
TGAACGGACACGAGTTCGAGATCGAGGGAGAGGGAGAGGGACGTCCATACGAGGGAACCCAAAC
CGCCAAGCTCAAGGTCACCAAGgtaagtttaaacatatatatactaactaaccctgattattta
aattttcag**GGAGGACCACTCCCATTCTCCTGGGACATCCTCTCCCCACAATTCATGTACGGAT**
CCCGTGCCTTCATCAAGCACCCAGCCGACATCCCAGACTACTGGAAGCAATCCTTCCCAGAGGG
ATTCAAGTGGGAGCGTGTCATGATCTTCGAGGACGGAGGAACCGTCTCCGTCACCCAAGACACC
TCCCTCGAGGACGGAACCCTCATCTACAAGGTCAAGCTCCGTGGAGGAAACTTCCCACCAGACG
GACCAGTCATGCAAAAGCGTACCATGGGATGGGAGGCCTCCACCGAGCGTCTCTACCCAGAGGA

bioRxiv preprint doi: https://doi.org/10.1101/2024.06.11.598534; this version posted June 13, 2024. The copyright holder for this preprint (which was not certified by peer review) is the author/funder, who has granted bioRxiv a license to display the preprint in perpetuity. It is made available under aCC-BY 4.0 International license.

CGTCGTCCTCAAGgtaagtttaaacagttcggtactaactaaccatacatatttaattttcag
GGAGACATCAAGATGGCCCTCCGTCTCAAGGACGGAGGACGTTACCTCGCCGACTTCAAGACCA
CCTACAAGGCCAAGAAGCCAGTCCAAATGCCAGGAGCCTTCAACATCGACCGTAAGCTCGACAT
CACCTCCCACAACGAGGACTACACCGTCGTCGAGCAATACGAGCGTTCCGTCGCCCGTCACTCC
ACCGGAGGATCCGGAGGATCCTAA

>wrmScarlet
GTCAGCAAGGGAGAGGCAGTTATCAAGGAGTTCATGCGTTTCAAGGTCCACATGGAGGGATCCA
TGAACGGACACGAGTTCGAGATCGAGGGAGAGGGAGAGGGACGTCCATACGAGGGAACCCAAAC
CGCCAAGCTCAAGGTCACCAAGGGAGGACCACTCCCATTCTCCTGGGACATCCTCTCCCCACAA
TTCATGTACGGATCCCGTGCCTTCACCAAGCACCCAGCCGACATCCCAGACTACTACAAGCAAT
CCTTCCCAGAGGGATTCAAGTGGGAGCGTGTCATGAACTTCGAGGACGGAGGAGCCGTCACCGT
CACCCAAGACACCTCCCTCGAGGACGGAACCCTCATCTACAAGGTCAAGCTCCGTGGAACCAAC
TTCCCACCAGACGGACCAGTCATGCAAAAGAAGACCATGGGATGGGAGGCCTCCACCGAGCGTC
TCTACCCAGAGGACGGAGTCCTCAAGGGAGACATCAAGATGGCCCTCCGTCTCAAGGACGGAGG
ACGTTACCTCGCCGACTTCAAGACCACCTACAAGGCCAAGAAGCCAGTCCAAATGCCAGGAGCC
TACAACGTCGACCGTAAGCTCGACATCACCTCCCACAACGAGGACTACACCGTCGTCGAGCAAT
ACGAGCGTTCCGAGGGACGTCACTCCACCGGAGGAATGGACGAGCTCTACAAG

(no start or stop included because this sequence was inserted at the C-terminus, before the stop codon, of the golg-4 gene)

>GFP (from pPD95.75)
ATGAGTAAAGGAGAAGAACTTTTCACTGGAGTTGTCCCAATTCTTGTTGAATTAGATGGTGATG
TTAATGGGCACAAATTTTCTGTCAGTGGAGAGGGTGAAGGTGATGCAACATACGGAAAACTTAC
CCTTAAATTTATTTGCACTACTGGAAAACTACCTGTTCCATGGgtaagtttaaacatatatata
ctaactaaccctgattatttaaattttcag**CCAACACTTGTCACTACTTTCTGTTATGGTGTTC**
AATGCTTCTCGAGATACCCAGATCATATGAAACGGCATGACTTTTTCAAGAGTGCCATGCCCGA
AGGTTATGTACAGGAAAGAACTATATTTTTCAAAGATGACGGGAACTACAAGACACGTaagttt
aaacagttcggtactaactaaccatacatatttaaattttcaggt**GCTGAAGTCAAGTTTGAAG**
GTGATACCCTTGTTAATAGAATCGAGTTAAAAGGTATTGATTTTAAAGAAGATGGAAACATTCT
TGGACACAAATTGGAATACAACTATAACTCACACAATGTATACATCATGGCAGACAAACAAAAG
AATGGAATCAAAGTTgtaagtttaaacatgattttactaactaactaatctgatttaaattttc
ag**AACTTCAAAATTAGACACAACATTGAAGATGGAAGCGTTCAACTAGCAGACCATTATCAACA**
AAATACTCCAATTGGCGATGGCCCTGTCCTTTTACCAGACAACCATTACCTGTCCACACAATCT
GCCCTTTCGAAAGATCCCAACGAAAAGAGAGACCACATGGTCCTTCTTGAGTTTGTAACAGCTG
CTGGGATTACACATGGCATGGATGAACTATACAAATAG
